# Co-activator candidate interactions for orphan nuclear receptor *NR2E1*

**DOI:** 10.1186/s12864-016-3173-5

**Published:** 2016-10-26

**Authors:** Ximena Corso-Díaz, Charles N. de Leeuw, Vivian Alonso, Diana Melchers, Bibiana K. Y. Wong, René Houtman, Elizabeth M. Simpson

**Affiliations:** 1 Centre for Molecular Medicine and Therapeutics at the Child and Family Research Institute, University of British Columbia, Vancouver, BC V5Z 4H4 Canada; 2 Genetics Graduate Program, University of British Columbia, Vancouver, BC V6T 1Z2 Canada; 3 Department of Medical Genetics, University of British Columbia, Vancouver, BC V6T 1Z3 Canada; 4 PamGene International B.V., Den Bosch, The Netherlands; 5 Department of Psychiatry, University of British Columbia, Vancouver, BC V6T 2A1 Canada; 6 Department of Ophthalmology and Visual Science, University of British Columbia, Vancouver, BC V5Z 3N9 Canada

**Keywords:** NR2E1, MARCoNI, Peptide array, Co-regulator, Orphan nuclear receptor

## Abstract

**Background:**

NR2E1 (Tlx) is an orphan nuclear receptor that regulates the maintenance and self-renewal of neural stem cells, and promotes tumourigenesis. *Nr2e1*-null mice exhibit reduced cortical and limbic structures and pronounced retinal dystrophy. NR2E1 functions mainly as a repressor of gene transcription in association with the co-repressors atrophin-1, LSD1, HDAC and BCL11A. Recent evidence suggests that NR2E1 also acts as an activator of gene transcription. However, co-activator complexes that interact with NR2E1 have not yet been identified. In order to find potential novel co-regulators for NR2E1, we used a microarray assay for real-time analysis of co-regulator–nuclear receptor interaction (MARCoNI) that contains peptides representing interaction motifs from potential co-regulatory proteins, including known co-activator nuclear receptor box sequences (LxxLL motif).

**Results:**

We found that NR2E1 binds strongly to an atrophin-1 peptide (Atro box) used as positive control and to 19 other peptides that constitute candidate NR2E1 partners. Two of these proteins, p300 and androgen receptor (AR), were further validated by reciprocal pull-down assays. The specificity of NR2E1 binding to peptides in the array was evaluated using two single amino acid variants, R274G and R276Q, which disrupted the majority of the binding interactions observed with wild-type NR2E1. The decreased binding affinity of these variants to co-regulators was further validated by pull-down assays using atrophin1 as bait. Despite the high conservation of arginine 274 in vertebrates, its reduced interactions with co-regulators were not significant *in vivo* as determined by retinal phenotype analysis in single-copy *Nr2e1*-null mice carrying the variant R274G.

**Conclusions:**

We showed that MARCoNI is a specific assay to test interactions of NR2E1 with candidate co-regulators. In this way, we unveiled 19 potential co-regulator partners for NR2E1, including eight co-activators. All the candidates here identified need to be further validated using *in vitro* and *in vivo* models. This assay was sensitive to point mutations in NR2E1 ligand binding domain making it useful to identify mutations and/or small molecules that alter binding of NR2E1 to protein partners.

**Electronic supplementary material:**

The online version of this article (doi:10.1186/s12864-016-3173-5) contains supplementary material, which is available to authorized users.

## Background

NR2E1 (Tlx) is an orphan nuclear receptor that regulates neural stem cell maintenance and self-renewal [1, 2] and promotes tumourigenesis [3, 4]. NR2E1 is crucial for adult stem cell proliferation [5] and plays an important role in spatial learning through promoting hippocampal neurogenesis [6]. *Nr2e1*-null mice are highly aggressive, have reduced cortical thickness and limbic structures [7], and display aberrant retinal development [8–10]. However, despite increased understanding regarding NR2E1 biological function and its gene targets [1, 11, 12] over the past few years, its precise molecular mechanism of action remains poorly understood.

Nuclear receptors interact with multi-protein co-activator or co-repressor complexes to activate or repress transcription, respectively. These co-regulator complexes have histone modification and chromatin remodeling functions that elicit transcriptional control. Many co-regulators interact with the hydrophobic groove of nuclear receptors through specific motifs. Co-repressors use the consensus amino acid sequence LxxxIxxxL or co-repressor nuclear receptor (CoRNR) box motif [13], while co-activators utilize an LxxLL binding motif, also called the nuclear receptor box [14].

NR2E1 functions mainly as a repressor of gene transcription and interacts with the co-repressors atrophin-1 (ATN1) [12], histone demethylase 1 (LSD1) [15], histone deacetylases (HDACs) 3, 5 and 7 [2], and the oncoprotein BCL11A [16]. In addition, NR2E1 can act as an activator of gene transcription by binding to the promoters of *Wnt7a* [1], and *Mash1* [17]. Despite all this knowledge, the composition and dynamics of the co-repressor complexes formed by NR2E1 are not well understood and, to date, no co-activator proteins have been found to interact with NR2E1. The crystal structure of NR2E1 in complex with a short sequence from atrophins (Atro box) was recently resolved showing that the autorepressed conformation of NR2E1 creates a pocket to recruit atrophins, which bind to NR2E1 through an unconventional sequence: ALxxLxxY [18].

To test whether NR2E1 could also bind to co-activators, we used a microarray assay for real-time analysis of co-regulator–nuclear receptor interaction (MARCoNI) [19] containing peptides representing co-regulator interacting sequences for which the LxxLL motif was highly enriched. Although many different approaches to characterize protein-protein interactions have been developed including far-western, yeast-two-hybrid, electrophoretic Mobility Shift Assays (EMSAs), and affinity purification followed by mass spectrometry, the MARCoNI array has the advantages of being high-throughput and allowing for rapid testing of small molecules or mutants that could affect the interaction of a nuclear receptor with its protein partners.

We confirmed the specificity of binding of NR2E1 to peptides in the array by using two single-point variants in the ligand binding domain: R274G and R276Q. We also evaluated whether R274G had an overt effect on retinal development in a mouse model carrying this variant as a single copy insertion.

## Results

To find novel transcriptional co-regulators that interact with NR2E1, we incubated the N-terminal FLAG-tagged ligand-binding domain (FLAG-NR2E1_LBD_) with peptides of a microarray assay for real-time analysis of co-regulator–nuclear receptor interaction (MARCoNI) (PamGene International). This array contained 154 peptides from 64 co-regulator proteins. Many of these peptides harbored the LxxLL motif. Since the array did not contain any previously known direct interactors for NR2E1, we added a peptide from atrophin-1 as a positive control. This peptide is referred to as the Atro box and is comprised of 16 amino acids that are highly conserved among atrophins and found to be necessary for the interaction between NR2E1 and atrophin-1 [20]. Two additional amino acids on each side were included for stability in the array (PYADTPALRQLSEYARPHVAFS). Mutations in the two leucines of the Atro box to alanines (PYADTPA**A**RQ**A**SEYARPHVAFS) abolish the interaction between *Drosophila* Atro and NR2E1 in yeast-two-hybrid assays [20]. Therefore, we included an Atro box containing these two leucine to-alanine substitutions as a negative control.

**Fig. 1:**
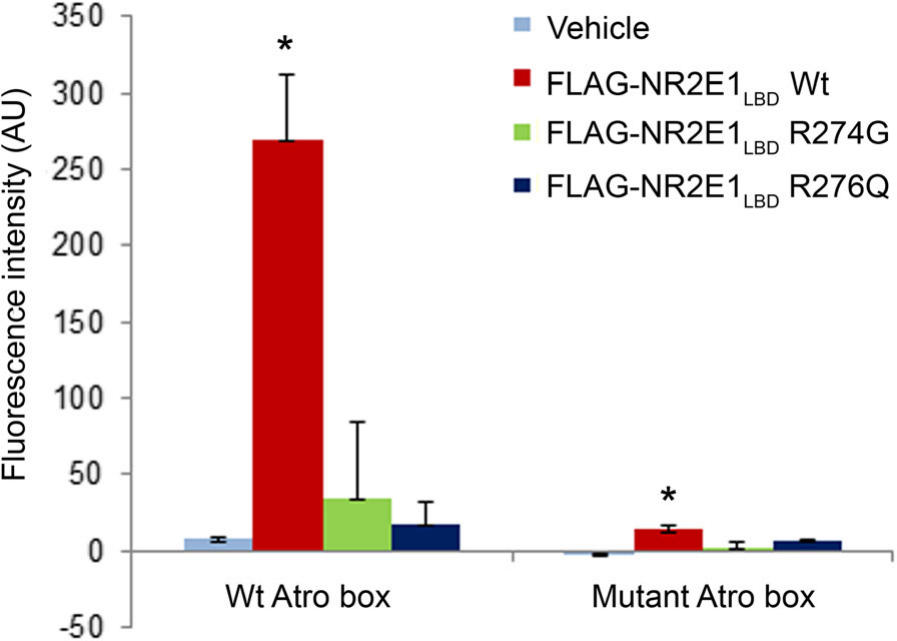
MARCoNI array validated with Atro box. Cell lysates from HEK293 cells expressing wild-type FLAG-NR2E1_LBD_ or variant FLAG-NR2E1_LBD_ (R274G or R276Q) protein truncations were prepared. Vehicle-treated cells were also included. These lysates were tested in the MARCoNI array, which contained a wild-type (PYADTPALRQLSEYARPHVAFS) and mutant (PYADTPAARQASEYARPHVAFS) Atro box as positive and negative controls, respectively. FLAG-NR2E1_LBD_ interacted strongly with the wild-type Atro box and with significantly reduced affinity to the mutant Atro box. The FLAG-NR2E1_LDB_ variants did not bind to either of the ATRO peptides. AU, arbitrary units; LBD, ligand binding domain; *, significantly different from wild-type (*p* < 0.01). *n* = 3

We found that FLAG-NR2E1_LBD_ interacted very strongly with the Atro-box peptide in the array but bound very poorly to the mutant Atro box (Fig. 1; *p* < 0.05, *n* = 3 arrays). This suggests that the array binding conditions are permissive for NR2E1 protein:protein interactions, and therefore appropriate for identifying novel interactions between NR2E1 and co-regulators.

**Fig. 2:**
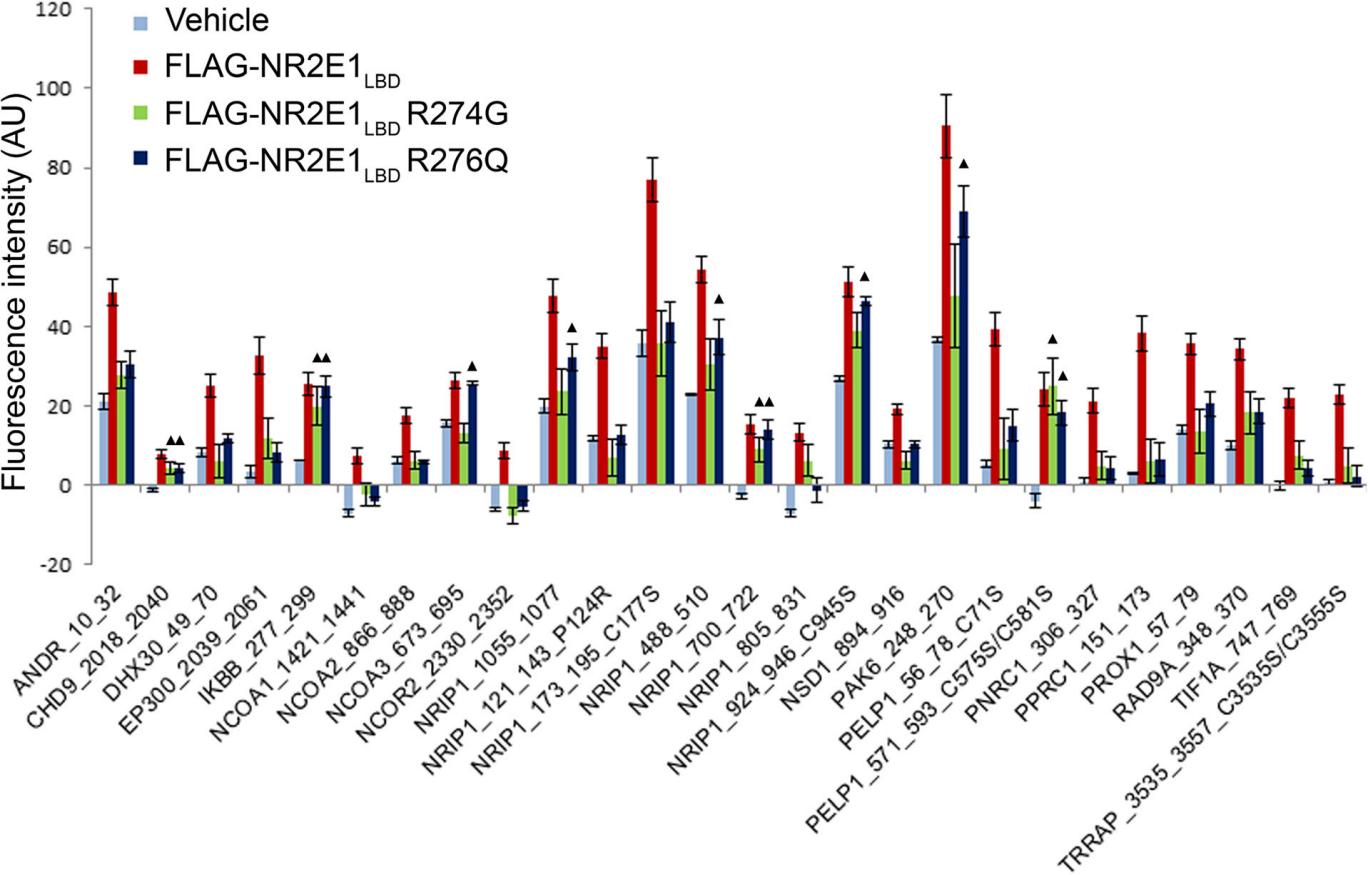
Twenty six novel co-regulator interacting peptides identified as binding to NR2E1 LBD in the MARCoNI array. Cell lysates from HEK293 cells expressing wild-type FLAG-NR2E1_LBD_ or variant FLAG-NR2E1_LBD_ (R274G or R276Q) protein truncations were prepared. Vehicle-treated cells were also included. These lysates were tested in the MARCoNI array, which contained 154 peptides from 64 co-regulator proteins. Only the significant FLAG-NR2E1_LBD_ binding interactions with 26 co-regulators (*q* < 0.05) are depicted in the histogram. The NR2E1_LBD_ variants abolished the majority of binding interactions observed between wild-type NR2E1_LBD_ and co-regulator peptides. However, triangles indicate peptides that did not interact significantly differently with NR2E1_LBD_ wild-type and the variant(s) (*q* > 0.05). AU, arbitrary units; LBD, ligand binding domain; peptide ID: co-regulator UniProt ID_amino acid start_amino acid end

FLAG-NR2E1_LBD_ also bound to 26 other peptides belonging to 19 proteins after applying False Discovery Rate correction for array size (*q* < 0.05) and eliminating negative fluorescence values (Additional file 1: Table S1 and Fig. 2). These interactions were; however, weaker than the interaction with atrophin-1 (Fig. 1) and may represent indirect interactions. Importantly, none of these proteins have previously been shown to directly or indirectly bind to NR2E1.

Some of the peptides that interacted with FLAG-NR2E_LBD_ in the array are cofactors known to function primarily in co-activator complexes. These include the SRC family members NCOA1 (SRC-1), NCOA2 (SRC-2) and NCOA3 (SRC-3) [21]; the ATP-dependent chromatin remodeling protein, Chromodomain Helicase DNA Binding Protein 9 (CHD9) [22]; Proline-Rich Nuclear Receptor Coactivator (PNRC1) [23]; Peroxisome Proliferator-Activated Receptor Gamma, Coactivator-Related (PPRC1) [24]; the scaffold Transformation/Transcription Domain-Associated Protein (TRRAP) [25] and the histone acetyltransferase P300 [26] (Fig. 2).

Other proteins that showed significant binding to FLAG-NR2E_LBD_ in the array are known to be primarily part of co-repressor complexes. These include the cell-cycle checkpoint protein human homolog of RAD9 [27]; the NR2E3 co-repressor and RNA helicase DHX30 (RetCoR) [28]; the HDAC-associated protein NCOR2 (SMRT) [29]; and Nuclear Receptor Interacting Protein 1 (NRIP1; RIP140) [30]. Interestingly, we found that six out of 14 NRIP1-derived peptides representing different motifs in the array showed significant interaction with FLAG-NR2E_LBD_ (Fig. 2).

We also observed that other co-regulators that can act as either co-activators or co-repressors are also targets in the array. These proteins are Proline, Glutamate and Leucine Rich Protein 1 (PELP1) [31]; the Histone methyltransferase Nuclear Receptor Binding SET Domain Protein 1 (NSD1) [32], and Tripartite Motif Containing 24 (TIF1A) [33] (Fig. 2).

In addition, transcription factors such as Prospero Homeobox 1 (PROX1) and androgen receptor (AR) also interacted with FLAG-NR2E_LBD_ in the array (Fig. 2). PROX1 functions as a co-regulator through LxxLL motifs [34] and AR harbors LxxLL-like motifs [35].

Other proteins that are sufficient to inhibit transcription factors were also candidate partners for NR2E1. These include P21 Protein (Cdc42/Rac)-Activated Kinase 6 (PAK6), which phosphorylates AR and inhibits its transcriptional activity [36]; and Nuclear Factor of Kappa Light Polypeptide Gene Enhancer in B-Cells (IKBB), that inhibits NF-kappa-B by sequestering it in the cytoplasm [37] (Fig. 2).

**Fig. 3:**
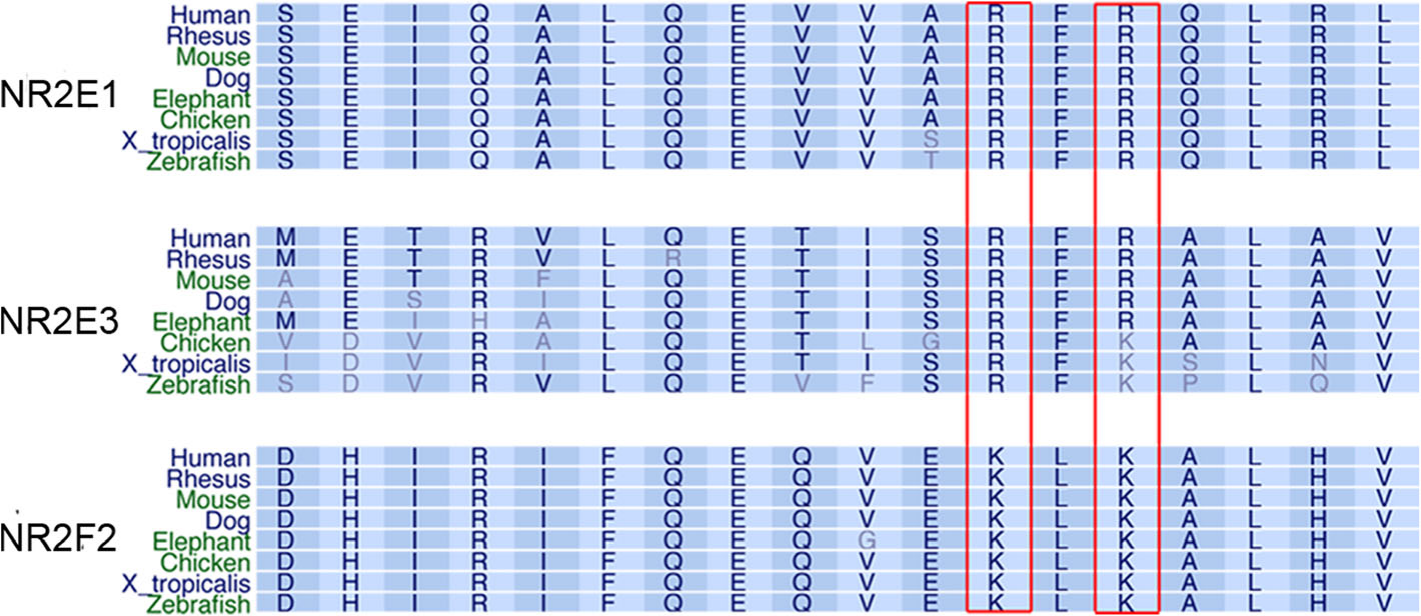
Arginines 274 and 276 are highly conserved in NR2E1. UCSC genome browser (https://genome.ucsc.edu/) image depicting NR2E1, and the conservation region of the two most related nuclear receptor binding domains; NR2E3 and NR2F2. A positively charged amino acid is conserved at positions 274 and 276. NR2E1 shows complete identity of arginine 274 and 276 from humans to zebrafish (red rectangles). In NR2E3, 274 is similarly conserved, but 276 becomes lysine in distant species. In NR2F2 a lysine, instead of arginine, is similarly conserved at both positions

To further assess the specificity of the array, we developed two FLAG-NR2E1 variants, each harboring a single-point variant in the LBD of NR2E1: an arginine-to-glycine substitution in codon 274 (R274G) and an arginine-to-glutamine substitution in codon 276 (R276Q). The LBD of NR2E1 is highly conserved in vertebrates (Fig. 3), so we hypothesized that amino-acid changes in the LBD will affect the ability of NR2E1 to interact with its protein partners. We previously identified an amino-acid change of unknown significance in arginine 274-to-glycine in the human population (mother and son) in the heterozygous state [38]. This arginine is highly conserved not only within NR2E1 but also within its close relative NR2E3. Furthermore, amino-acid similarity is also found in other nuclear receptors of the same family such as NR2F2 where there is a lysine instead of an arginine in aligned regions of the ligand binding domain corresponding to NR2E1 codon 274 (Fig. 3). This conservation suggests that an amino-acid with a positively-charged side chain in this region is important for normal protein function. Furthermore, arginine 274 corresponds to arginine 309 in NR2E3 and its substitution to glycine in NR2E3 affects photoreceptor development in humans [39, 40]. Interestingly, we observed that both variants caused a reduction in the binding of NR2E1 to the Atro box and the majority of the peptide interactions found in the array (17/26 peptides). The exceptions were peptides belonging to PELP1, IKBB, CDH9 and NRIP1 which did not show a significant reduction in interaction with FLAG-NR2E1_LBD_ R274G and R276Q, and also PAK6 and NCOA3, which showed comparable binding to FLAG-NR2E1_LBD_ R276Q and wild-type NR2E1, but reduced binding to FLAG-NR2E1_LBD_ R274G (Fig. 2).

**Fig. 4:**
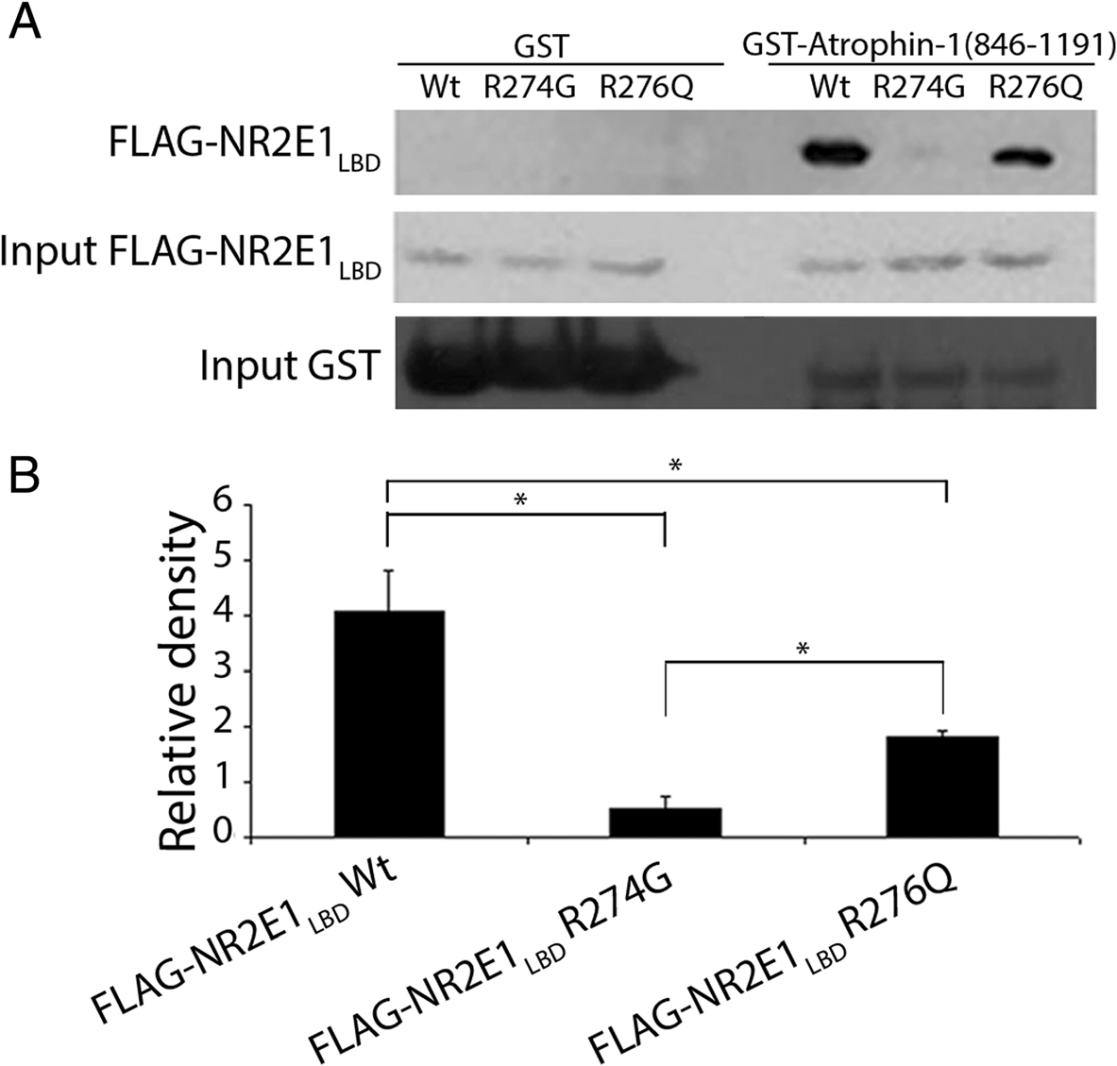
NR2E1 variants R274G and R276G exhibited decreased binding to atrophin-1. GST, or GST-Atrophin-1_846–1191_ containing the Atro box, were over-expressed in *E.coli* and incubated with HEK293 cell lysate containing either FLAG-NR2E1_LBD_, FLAG-NR2E1_LBD_ R274G, or FLAG-NR2E1_LBD_ R276Q. After incubation with glutathione/sepharose beads, purified complexes were resolved by SDS-PAGE followed by western blot using an anti-FLAG antibody. **a** Note the decreased binding of the FLAG-NR2E1_LBD_ R274G and R276Q variants to GST-Arophin-1_846–1191_. **b** Western blot quantification of three independent experiments. The signal intensity detected with anti-FLAG antibody for each pull down was normalized to the input signal. Note that wild-type NR2E1 binds eight times more to atrophin-1 compared to R274G. Error bars, standard error of the mean; GST, glutathione-S-transferase; LBD, ligand binding domain FLAG. *n* = 3

We further studied the ability of FLAG-NR2E1_LBD_ R274G or R276Q to interact with human atrophin-1 by affinity purification assays. We used bacterially expressed atrophin-1-GST (846–1191) containing the Atro box as a bait and incubated it with HEK293 lysates containing the wild-type and variant FLAG-NR2E1_LBD_ proteins used in the peptide array. In agreement with what we observed in the array, there was decreased binding of both R274G and R276Q to atrophin-1 suggesting that these amino acid changes alter the conformation of NR2E1 and its capacity to interact with its protein partners (Additional file 2: Table S2 and Fig. 4).

**Fig. 5:**
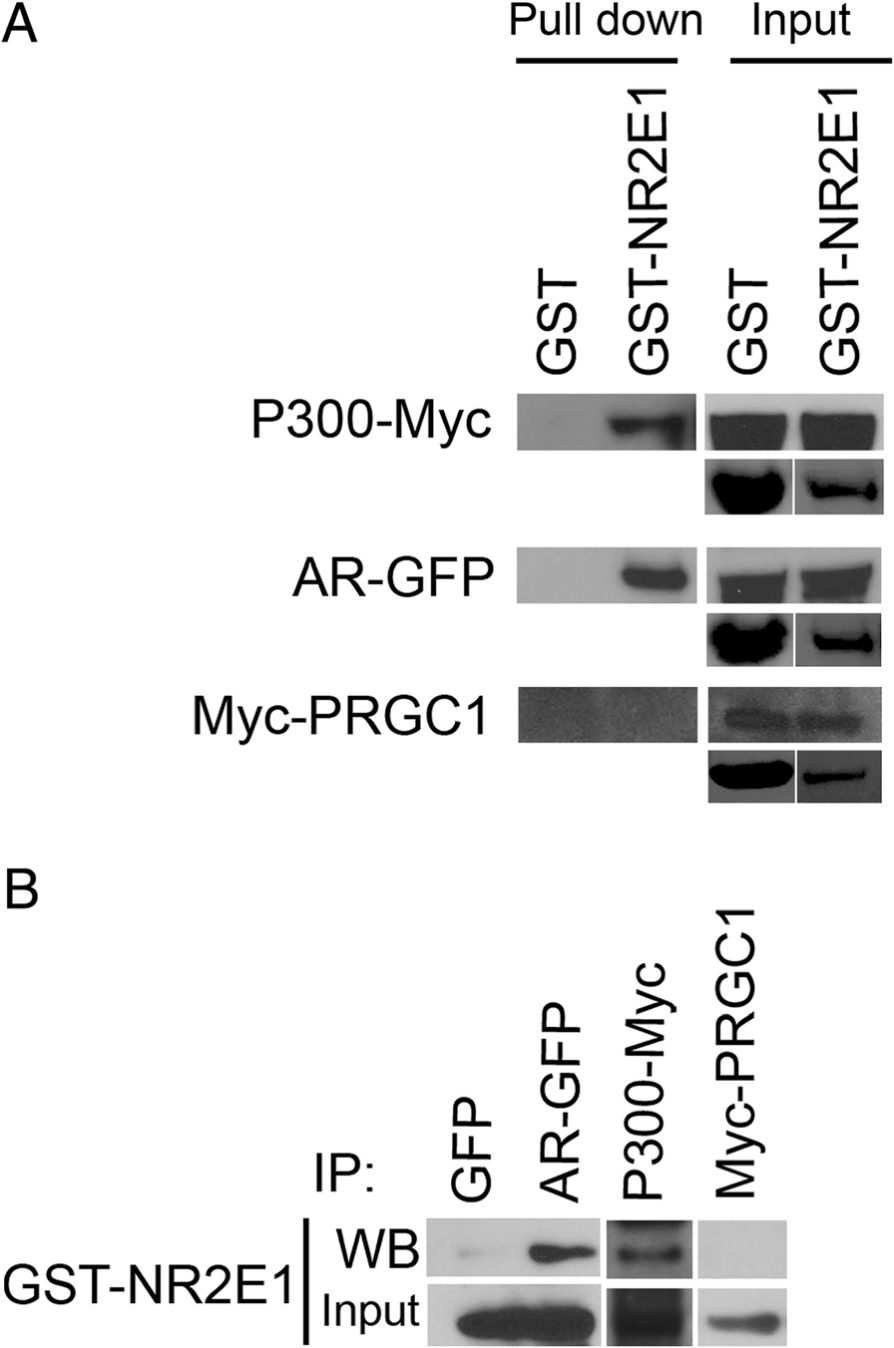
NR2E1 interacted with P300 and androgen receptor. GST, or GST-NR2E1_LBD_ were over-expressed in *E.coli* and incubated with HEK293 cell lysate containing different overexpressed proteins: (**a**) Myc-tagged P300 (P300-Myc), EGFP-tagged androgen receptor (AR-EGFP) and myc-tagged PRGC1 (Myc-PRGC1). After incubation with glutathione/sepharose beads, purified complexes were resolved by SDS-PAGE followed by western blot using anti-EGFP, and anti-myc antibodies. Note that P300 and AR do not bind to the GST control but are pulled-down with NR2E1-GST. Also note the absence of binding of PRGC1 to both GST control and GST-NR2E1. GST input represents images of Ponceau S-stained blots that were converted to black and white. **b** Immunoprecipitation was carried out using anti-P300, anti-EGPP, and anti-Myc antibodies. Complexes were resolved by SDS-PAGE followed by western blot. GST-NR2E1 was detected using anti-GST antibody. Note the binding of GST-NR2E1 to P300 and AR but not to PRGC1. GST, glutathione-S-transferase; IP, immunoprecipitation; WB, western blot

We selected two proteins, AR and P300, from the array for further analysis. AR is a nuclear hormone receptor of the NR3C class. We chose AR because in addition to its well-known role in cancer and male reproductive function, it also has a role in neurogenesis [41–43]. The array included the FxxLF motif of AR, located in its NH_2_-terminal region. This motif interacts with AR itself to stabilize the ligand-bound AR complex by interacting with different regions of the AR ligand binding domain [35]. The FxxLF motif also mediates AR interaction with co-regulators such as Melanoma Antigen Gene Protein-A11 (MAGE-11) [44]. P300 is an acetyltransferase that acetylates histones and other proteins. P300 also acts as a scaffold for transcription factors and other components of the basal transcription machinery to facilitate chromatin remodeling and gene transcription [26]. We chose P300 because it is a co-activator that has well-established roles in regulating neural stem cell differentiation [45, 46]. We observed that AR and P300 precipitated with NR2E1 in reciprocal pull-downs (Fig. 5). Importantly, we also verified the lack of binding of PRGC1 in the MARCoNI array by pull-down experiments (Fig. 5).

**Fig. 6:**
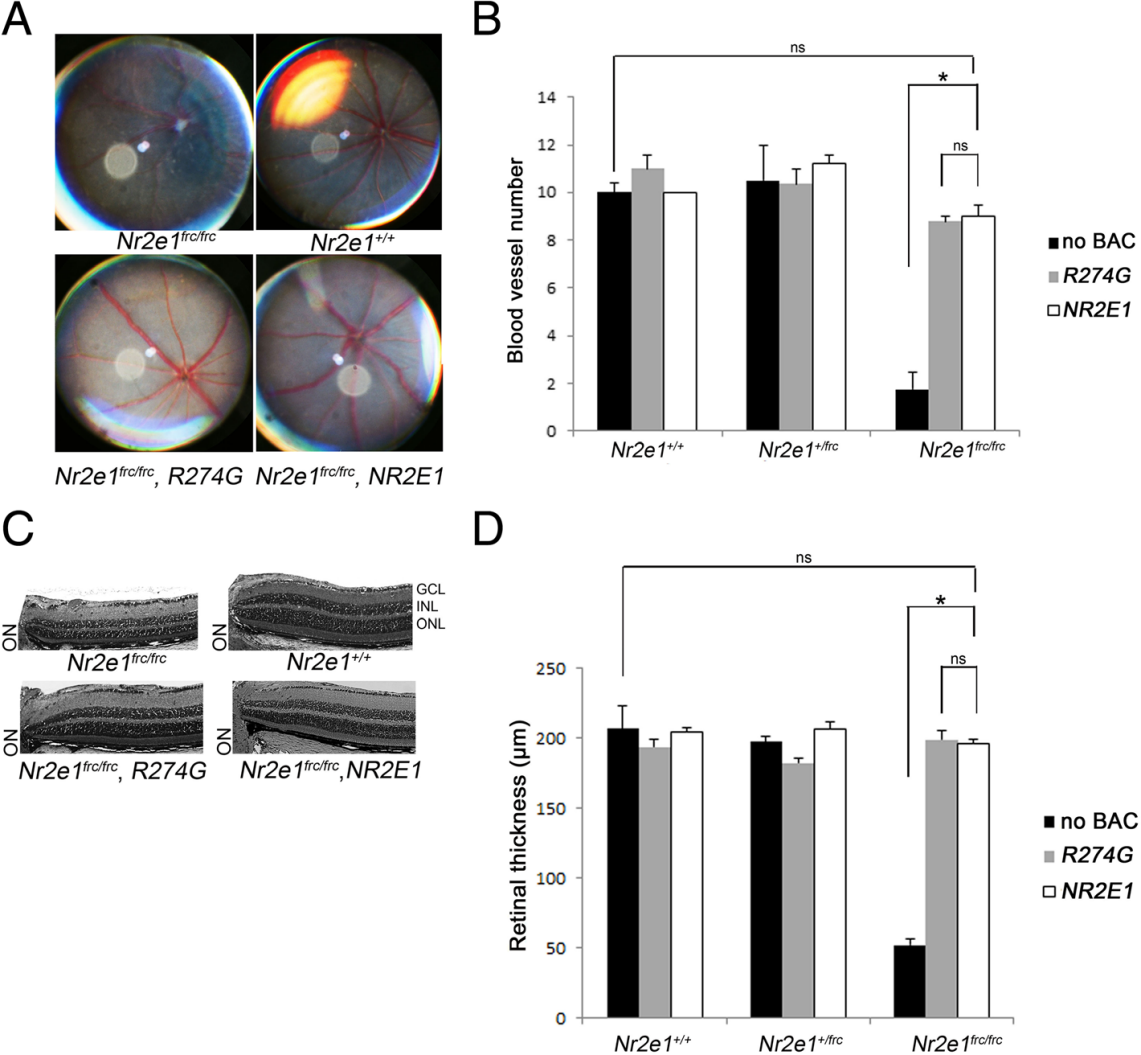
The variant R274G rescued retinal blood vessel numbers and thickness. P28 *Nr2e1* ^*+/+*^ and *Nr2e1* ^*frc/frc*^ controls, and *Nr2e1* ^*frc/frc*^ mice harboring a human *NR2E1* or *R274G* BAC single-copy knock-in at the *Hprt* locus were studied. **a** Fundus pictures showing reduced blood vessel numbers and abnormal morphology of *Nr2e1* ^*frc/frc*^ mice, similar to previous reports [10, 47], and apparent rescue of this defect by both *NR2E1* and the variant *R274G*. **b** Quantification of retinal blood vessels showing a reduction in *Nr2e1* ^*frc/frc*^ retinas compared to wild-type numbers. *NR2E1* and the variant *R274G* rescued this defect. **c** Paraffin-embedded eyes were sectioned at 5 μm thickness. Central sections containing the optic nerve were stained with H and E. Representative retinal pictures are depicted showing very thin retinas in *Nr2e1* ^*frc/frc*^ mice and apparent rescue of this defect by *NR2E1* and the variant *R274G*. **d** Quantification of retinal thickness showed a high reduction in *Nr2e1* ^*frc/frc*^ retinas compared to wild-type and a rescue of this defect by *NR2E1* and the variant *R274G*. Error bars, standard error of the mean; GCL, ganglion cell layer; INL, inner nuclear layer; ns, no significant; ON, optic nerve; ONL, outer nuclear layer; P#, postnatal day #; *, *p* < 0.001. *n* = 6 (12 eyes)

Since the NR2E1 variants used in this study have a dramatic effect on the ability of NR2E1 to interact with its partners, we studied the effects of R274G during retinogenesis *in vivo*. We utilized the eye due to its salient and robust phenotype in *Nr2e1*-null (*Nr2e1* ^*frc*/*frc*^) mice of retinal disorganization and abnormal blood vessels, as well as for the feasibility of studying eye defects. We have previously shown that a wild-type copy of human *NR2E1* can rescue the *Nr2e1*-null phenotype in the eye [47]. We analyzed the retinal blood vessel numbers and retinal thickness of a transgenic mouse harboring the R274G mutation as a single-copy on the X-Chromosome [48]. We compared littermates that were wild-type, *Nr2e1* ^*frc/+*^ or *Nr2e1* ^*frc/frc*^, with or without the single-copy *Hprt* knock-in R274G variant. In this way, we could study possible gain of function, dominant negative or loss of function behavior of R274G. Strikingly, we found that the variant R274G did not affect blood vessel or radial symmetry (Fig. 6a and b). Similarly, the reduced retinal thickness of *Nr2e1* ^*frc/frc*^ mice was not observed in mice harboring the R274G variant on any of the *Nr2e1* backgrounds studied (Fig. 6c and d), suggesting the ability to functionally act as wild-type. In conclusion, R274G does not generate an overt retinal phenotype suggesting that there are compensatory mechanisms that rescue the reduced NR2E1 binding observed *in vitro*.

## Discussion

In this study, we used a peptide array to find novel interactors for NR2E1 that revealed 19 candidate co-regulator proteins. We also found two single amino-acid variants of NR2E1 that disrupted its binding to most co-regulators and that one of them, R274G, did not have an overt physiological significance in the retina. Our results identify putative co-regulators that can interact with NR2E1.

Proteins interact through domains, many of which bind to short peptide sequences [49]. Although the peptides in the array represent some of these short linear sequences, they lack the context of the full protein and thus may participate in non-specific interactions. To evaluate the specificity of the array and the appropriateness of this approach, we used as a positive control the Atro box, and as a negative internal control the mutated Atro box. We also used the FLAG-NR2E1_LBD_ single amino-acid variants R274G and R276Q as experimental negative controls. We observed strong binding of NR2E1 to the wild-type Atro box and decreased binding to the mutated Atro box. Furthermore, we observed that FLAG-NR2E1_LBD_ R274G and R276Q failed to interact with most of the NR2E1 putative interactors in the array suggesting that these variants generated a conformational change in NR2E1 that prevented its binding to other proteins. This hypothesis was further strengthened in pull-down assays using atrophin-1 as bait where both NR2E1 variants interacted with atrophin-1 with much less affinity than with wild-type. Together, these data suggest that the binding of NR2E1 to the interactors in the array was specific.

Importantly, we noted that the binding of NR2E1 to other peptides in the array was weaker than the binding observed for atrophin-1, a known direct NR2E1 interactor. This could be due to the fact that the interaction of NR2E1 to the other peptides in the array is more transient or indirect.

Our results then suggest that NR2E1 uses different co-regulator complexes to affect neural precursor behavior. For example, mouse Prox1, an interactor of NR2E1 in the array, has a role in controlling the balance between neural progenitor self-renewal and neuronal differentiation by inhibiting Notch1 expression [50]. Interestingly, the expression of the *Prox1* homolog in *Drosophila*, *prospero*, is regulated by the homolog of *NR2E1*, *Tlx* [51], suggesting a role for NR2E1 in controlling PROX1 biological output. Another interactor in the array that is also a neural precursor-related protein is DHX30 (RetCoR), which is important for mediating NR2E3 repressor function in retinal progenitor cells [28].

An intriguing co-repressor target is NRIP1 that had 14 motifs in the array, out of which six interacted with NR2E1. NRIP1 is expressed in the brain including the neurogenic region of the dentate gyrus [52], but its role in neurogenesis, if any, is unknown and worth exploring.

Interestingly, three co-activators members of the SRC family (SRC-1, SRC-2 and SRC-3) were found to interact with NR2E1 in the array. Although it is unknown whether they have a role in neural stem cell behavior, there is some evidence that SRC-1 could be involved in neurogenesis as it is upregulated during neuronal differentiation *in vitro* [53].

Two important scaffolds of multi-protein co-activator complexes, TRRAP and P300, also interacted with NR2E1 in the array. TRRAP is found mutated in patients with schizophrenia [54] and controls the tumourigenicity of brain tumour initiating cells which resemble neural stem cells [55]. In the brain, P300 mediates the induction of the astrocyte lineage [46] and works with the transcription factor Zfp521 to directly activate early neural genes [45]. Understanding the biological importance of the interaction of these adaptor proteins with NR2E1 would greatly advance our knowledge of NR2E1 mechanisms of action.

Many nuclear receptors have been shown to cooperate to regulate gene transcription. It is an interesting possibility that AR and NR2E1 could function in a complex to synergistically regulate adult neurogenesis where testosterone plays a role [42, 43]. In addition, this interaction could be important during the development of prostate cancer where NR2E1 has been shown to have an oncogenic function [4].

Importantly, we successfully pulled-down P300 and AR with GST-NR2E1, thus reproducing by an orthogonal experiment the results from the array. This strongly suggests that the array is a reliable tool to identify novel nuclear receptor interactors.

Intriguingly, the variant R274G did not have an effect on retinal development *in vivo*. Since we observed decreased binding of R274G to atrophin-1 in two orthogonal approaches, we suggest that NR2E1 protein conformation was indeed compromised by this arginine-to-glycine substitution. It is possible that the interactions detected in this study are not functional during gross retinal development or that there may have been a compensatory mechanism such as upregulation of NR2E1 or some of its co-regulators to compensate for the lower affinity of NR2E1 towards its protein partners. Alternatively, since we only used the ligand binding domain of NR2E1 in the array whereas our mouse strains express the full length protein (wild-type or variant), it is possible that the NR2E1-co-regulator complexes are more stable in the full length conformation. In this way, some co-regulators could bind to NR2E1 through both its LBD and DBD as is the case for LSD1 [15]. It is also possible that variant R274G could have an effect on brain development. However, we could not assess this phenotype as the BAC we use in this study does not completely rescue the brain abnormalities observed in *Nr2e* ^*−/−*^ [47].

The mouse model harboring the variant R274G validates again the usefulness of our cloning strategy showing that a single-copy human gene incorporated into the *Hprt* locus is functional. This strategy could be useful to study human mutations in the regulatory or coding regions of disease-causing genes.

Similar to COUP-TFII, which can be activated by retinoic acids despite its autorepressed conformation [56], NR2E1 can be regulated by artificial ligands [57]. Therefore, a peptide array could represent a rapid screening tool for small molecules that modify the ability of NR2E1 to bind co-regulators.

## Conclusion

By using a highly specific peptide array, we uncovered novel putative interactors for NR2E1 including the co-activator P300 and the nuclear receptor AR. Future experiments will aim to validate the interactions here discovered and understand their biological significance. This array could be used to test small molecules and mutations that may alter the interaction of NR2E1 with co-regulators and therefore its transcriptional function.

## Methods

### Microarray assay for real-time analysis of co-regulator–nuclear receptor interaction (MARCoNI)

The peptide array assay was performed on Nuclear Receptor PamChip Arrays (PamGene International, B.V., Den Bosch, the Netherlands) harboring 154 immobilized peptides corresponding to co-regulator-derived NR interaction motifs (#88101; PamGene International[19]) as described before [58]. We added a peptide from *Drosophila* atrophin-1, called the Atro box (PYADTPALRQLSEYARPHVAFS) as a positive control and a mutant Atro box in which two leucines were substituted with alanines (PYADTPAARQASEYARPHVAFS) as a negative control [20]. Briefly, lysates were prepared from HEK293 cells transfected with the human NR2E1 ligand binding domain FLAG-tagged at the N-terminus (FLAG-NR2E1_LBD_), wild-type or containing the variants R274G or R276Q as described below. The concentration of FLAG-NR2E1_LBD_ in the lysates was assessed by western blot and the quantities of each sample were adjusted such that equal amounts of NR2E1 proteins were added to the array. Sample IDs were randomly assigned to allow for a blinded experiment. Anti-FLAG M2 monoclonal antibody-FITC conjugate (Sigma-Aldrich, St. Louis, MO, #F4049) at 1.1 mg/ml was added to the lysates to detect the overexpressed proteins.

The arrays were incubated with 25 μL blocking buffer (TBS with 1 % BSA, 0.01 %, Tween-20, and 0.3 % skimmed milk) for 20 cycles. Subsequently, each array was incubated with 7–10 μL of assay mix (TBS with 0.2 % BSA, 0.05 % Tween-20, 50 μM DTT, 2 % DMSO, 25 nM anti-Flag-FITC) for 120 cycles (30 s/cycle) and washed with 25 μL TBS. After washing an additional detection step was performed with 54 nM goat anti mouse FITC (Santa Cruz #SC-2010, 200 μg/ml) for 20 cycles and washed with 25 μl TBS. At cycle 163, a TIFF (Tagged Image File Format) image was captured by the CCD camera. Image analysis was performed by automated spot finding and quantification using BioNavigator software (PamGene International BV) as described before [58]. The background was discounted from the spot signal. Each immobilized peptide was quantitatively assessed for abundance, using Syproruby staining, and functionality, using a standard glucocorticoid receptor MARCoNI assay, which measures dexamethasone-induced modulation. Three technical replicates were performed.

### Plasmid constructs and site-directed mutagenesis

To make FLAG-NR2E1_LBD_, the NR2E1 ligand binding domain (LBD) was cloned into the pEGFP-N1 vector (Clontech, CA, USA) using *Eco*RI and *Not*I enzymes (Invitrogen, CA, USA). The EGFP coding region was replaced with NR2E1 LBD that was either wild-type or that contained the R274G or R276Q amino-acid changes. The NR2E1 LBD was amplified using the primers: forward 5′-ATATGAATTCACCATGGACTACAAGGATGACGATGACAAGGGAGGAGGAGGAGGAGGAGTGTCCACCACTCCAGAGCGGC-3′ and reverse 5′-ATATGGATCCTTAGATATCACTGGATTTGTAC-3′. The forward primer also contained the FLAG-tag coding sequence.

Site directed mutagenesis was performed with the Quik-Change® Lightning Site-Directed Mutagenesis Kit (Stratagene, CA, USA) on wild-type NR2E1 according to manufacturer’s instructions. Primers used were forward 5′-GAGGTGGTGGCTCGATTTCAACAACTCCGGTTAGATGC-3′ and reverse 5′-GCATCTAACCGGAGTTGTTGAAATCGAGCCACCACCTC-3′ for R274G; and forward 5′-GCTTTACAAGAGGTGGTGGCTGGATTTAGACAACTCC-3′ and reverse 5′-GGAGTTGTCTAAATCCAGCCACCACCACTTGTAAAGC-3′ for R276Q.

GST-NR2E1 was generated by cloning full-length NR2E1 into pGEX-2 T vector (Clontech, CA, USA) using *Bam*HI and *Eco*RI enzymes (Invitrogen, CA, USA). NR2E1 cDNA was amplified using the following primers: forward 5′-ATATGGATCCGGAGGAGGAGGAGGAGGAATGAGCAAGCCAGCC-3′ and reverse 5′-ATATGAATTCTTAGATATCACTGGATTTGTAC-3′.

pEGFP-C1-AR (Deposited by Michael Mancini [59], Plasmid #28235), pCMVβ-p300-myc (Deposited by Tso-Pang Yao, Plasmid #30489), and pcDNA4 myc-PGC-1 alpha (Deposited by Toren Finkel [60], Plasmid #10974) were purchased from Addgene (Cambridge, MA, USA). The GST human -Arophin-1_846–1191_ construct was kindly donated by Dr. Chih-Cheng Tsai (Baylor College of Medicine, Houston, TX, USA).

### Protein expression and protein lysates preparation

One Shot® BL21 Star™ (DE3) Chemically Competent *E. coli* (Life Technologies, Carlsbad, CA, USA) were transformed with GST-atrophin-1 or GST-NR2E1 constructs. Subsequently, a bacterial colony was grown in 10 mL of LB media containing 100 μg/mL Ampicillin overnight. Five μL of the overnight culture were added to 100 mL of LB (1:50 dilution) containing 100 μg/mL Ampicillin and grown to reach an OD_600_ of 0.5 (mid-log). Bacteria were induced with 0.1 mM IPTG for 4 h at 37 °C. Bacteria were pelleted at 7,000x *g* for 7 min, washed with STE buffer (10 mM Tris pH8.0, 150 mM NaCl and 1 mM EDTA) and resuspended in STE buffer containing protease inhibitors (Roche, Roche, Basel, Switzerland) and 100 μg/mL of lysozyme. Cells were incubated 15 min on ice and DTT was added to a final concentration of 5 mM. Subsequently, Sarkosyl was added to a final concentration of 0.2 %, and lysates were vortexed and sonicated on iced twice for 15 s each. Lysates were adjusted to 1 % Triton X-100 and centrifuged at 20,000x *g* for 15 min at 4 °C. Supernatants were collected and used in subsequent experiments.

To express FLAG-NR2E1_LBD_ variants, HEK293 cells were grown in T-75 flasks and transfected with each of the different constructs. Forty-eight hours after transfection, cells were washed with PBS and lysed in ice-cold solubilization buffer [20 mM Tris HCl, pH8.0, 1 % NP40, 10 % glycerol, and 137 mM NaCl] with protease inhibitor cocktail (Roche, Basel, Switzerland) and 1 mM phenylmethylsulfonyl fluoride (PMSF). Cells were sonicated for 10 s and centrifuged at 20,000x *g* for 10 min at 4 °C.

### Pull-down experiments

Bacterial lysates were incubated with glutathione MagBeads (GenScript, Piscataway, NJ, USA) for one hour. Beads were then washed twice with PBS and incubated in 1 % BSA/PBS for one additional hour. HEK293 lysates were adjusted to 0.1 % BSA and incubated with the beads for two hours at 4 °C. Beads were washed three times for 30 min before adding 50 μL of loading buffer. SDS-Polyacrylamide gel electrophoresis was performed with the final sample from the beads and 3 % of recovered input. For immunoprecipitation experiments, HEK293 lysates were precleared with protein A beads for 1 h and added to beads-antibody conjugates for an additional hour. After removing the supernatant, the second HEK293 lysate expressing the candidate interactor was added for 2 h. Beads were washed four times with solubilization buffer before proceeding to SDS-PAGE. Antibodies used were rabbit anti-P300 (Sydlabs, Natick, MA, USA), rabbit anti-EGFP (Invitrogen, Carlsbad, CA, USA), rabbit anti-Myc and rabbit anti-Flag (Sigma-Aldrich, St. Louis, MO, USA).

### Western blot

Samples containing 1X loading buffer were incubated at 75 °C for 10 min and loaded into NuPAGE® Novex Bis-Tris Gels (Invitrogen, Carlsbad, CA, USA). Gels were run for 30–45 min at 150 V. Subsequently transfer onto polyvinylidene difluoride (PVDF) membranes was performed for 90 min at 30 V. After transfer, membranes were washed and incubated in blocking solution (5 % milk in Tween-20-TBS) for one hour. Subsequently, membranes were incubated with primary antibodies in blocking solution overnight at 4 °C. Antibodies used were rabbit anti-P300 (Sydlabs, Natick, MA, USA), rabbit anti-EGFP (Invitrogen, Carlsbad, CA, USA), rat anti-GST, rabbit anti-Myc, and rabbit anti-Flag (Sigma-Aldrich, St. Louis, MO, USA). After three washes of 10 min each, membranes were incubated with peroxidase-conjugated secondary antibodies. After three subsequent washes, the membrane was incubated with Pierce™ ECL Chemiluminescent Substrate (Thermo Fisher Scientific Inc, Rockville, MD, USA) and exposed to Fuji Rx film.

### Mouse strains husbandry and breeding

B6.129P2(Cg)-*Hprt* ^*tm330(NR2E1,bEMS112)Ems*^ mice were generated from embryonic stem cells (ESCs) (clone mEMS4738) that harbored the bacterial artificial chromosome (BAC) bEMS112 (containing variant R274G) knock-in allele at the mouse *Hprt* locus, as previously described [47, 48]. Only male mice were studied to avoid variability due to random X inactivation of the knock-in allele at *Hprt*. Experimental animals for studying NR2E1 BAC bEMS112 were generated through a breeding strategy described before [47]. Briefly, B6 (C57BL/6 J) females heterozygous for the BAC insert (*N* > 10) and for the fierce deletion (B6.Cg-*Hprt* ^*tm85(NR2E1,bEMS112)Ems*^/X, *Nr2e1* ^*frc/+*^) were crossed to 129 (129S1/SvImJ) males heterozygous for the fierce mutation (129S1/SvImJ.Cg-*Nr2e1* ^*frc/+*^) (*N* > 10). This produced first-generation hybrid offspring (B6129F1), abbreviated here as *Nr2e1* ^*+/+*^; *Nr2e1* ^*frc/+*^; *Nr2e1* ^*frc/frc*^; *Nr2e1* ^*+/+*^ *, NR2E1*; *Nr2e1* ^*frc/+*^ *, NR2E1*; *Nr2e1* ^*frc/frc*^ *, NR2E1*; *Nr2e1* ^*+/+*^ *, R274G*; *Nr2e1* ^*frc/+*^ *, R274G*; and *Nr2e1* ^*frc/frc*^ *, R274G*. Mice were kept in a pathogen-free animal facility at the Centre for Molecular Medicine and Therapeutics (Vancouver, BC, Canada) on an 6 am to 8 pm light cycle at 20 ± 2 °C with 50 % ± 5 % relative humidity, and food and water *ad libitum*.

### Funduscopy

To assess the number of retinal blood vessels, funduscopy was performed using a Kowa Genesis small animal fundus camera (Pacific Medical, Delta, BC, Canada) as previously described [47, 61]. In short, eyes were dilated with 1 % atropine/PBS and photographed after 30 min. Animals were manually restrained without sedation.

### Histology

Eyes were dissected, washed once in PBS and incubated overnight in Davidson’s fixative. Eyes were stored in 70 % ethanol until paraffin embedment. Eyes were sectioned at 5 μm and mounted on SuperFrost Plus slides. To evaluate retinal thickness, eyes were subjected to hematoxylin and eosin staining. Briefly, tissue was incubated in hematoxylin for 5 min, washed in tap water and incubated in 1 % lithium carbonate solution for 30 s. After washing in tap water again, the tissue was incubated in acid alcohol (1 %) for 5 s followed by another tap water wash and incubation in eosin Y solution for 5 min. After a final tap water wash, tissue was dehydrated in a gradient series of ethanol and xylene before mounting for microscopy. Retinal sections were chosen such that they contained the optic nerve and retinal thickness was measured 600 μm away from the optic nerve using the software ImageJ [62]. One section per eye and two eyes per animal were studied. Retinal sections were imaged with the Olympus BX61 motorized microscope using the software DP Controller (Olympus, Tokyo, Japan).

### Statistical analysis

Statistical analysis was performed using the software XLSTAT. *T*-test was used to calculate differences between groups. False discovery rate (FDR) q-values were calculated using the Benjamini-Hochberg procedure to correct for multiple hypothesis testing [63]. A comparison between signal intensities in the western blot was performed by one-way ANOVA.

## Additional files

Additional file 1:
**Table S1**. List of all peptides used in the MARCoNI array and their corresponding binding values to NR2E1 Wt and variants R274G and R276Q. (XLS 48 kb)
Additional file 2:
**Table S2.** Densitometry analysis of western blot signal after pulling-down NR2E1 Wt and variants with GST-Atrophin-1. (XLSX 9 kb)
